# Combined Burden of Preterm and Low Birthweight Births in Greece: A Nationwide Time-Trend Analysis

**DOI:** 10.7759/cureus.95160

**Published:** 2025-10-22

**Authors:** Nikolaos Vlachadis, Dimos Sioutis, Chrissi Christodoulaki, Nikolaos Machairiotis, Charalampos Theofanakis, Konstantinos Louis, Periklis Panagopoulos

**Affiliations:** 1 Third Department of Obstetrics and Gynecology, National and Kapodistrian University of Athens, Medical School, Attikon University Hospital, Athens, GRC; 2 Department of Obstetrics and Gynecology, General Hospital of Chania, Chania, GRC

**Keywords:** greece, low birthweight, preterm and low birthweight infants, preterm births, small vulnerable newborns

## Abstract

Introduction: The objective of this study was to assess the combined burden of preterm and low birthweight (LBW) births in Greece from 1980 to 2023, using this measure as a proxy for small vulnerable newborns (SVN), the neonatal population at heightened risk of mortality and of both short- and long-term morbidity.

Materials and methods: The analysis included 4,585,090 live births in Greece with complete data from 1980 to 2023. Trends in preterm (< 37 weeks of gestation) and/or LBW (< 2,500 g) births, as well as in the three subcategories (preterm-LBW, preterm-non-LBW, and term-LBW), were assessed using Joinpoint regression analysis (Joinpoint Regression Program, version 5.2.0; National Cancer Institute, Bethesda, MD, USA). Annual percent change (APC) and 95% confidence intervals (CI) were estimated for each category.

Results: The prevalence of SVN declined significantly from 1980 to 1990 (APC = -1.8, 95% CI: -2.7 to -1.1, p < 0.001), reaching an all-time low of 6.98% in 1990. Thereafter, it rose to a historic peak of 15.28% in 2023. The main increase occurred during 1990-2010 (APC = 3.7, 95% CI: 3.5-4.0, p < 0.001), followed by a non-significant upward trend from 2010 to 2023. The proportion of preterm-LBW births increased from 1.69% in 1991 to a historic maximum of 7.14% in 2023. The steepest increases were observed between 1991 and 2006 (APC = 5.5, 95% CI: 3.8-13.5, p = 0.004) and 2006 and 2009 (APC = 14.1, 95% CI: 0.4-16.1, p = 0.038), continuing more slowly thereafter (2009-2023: APC = 1.3, 95% CI: 0.6-2.2, p = 0.026). The proportion of preterm-non-LBW neonates also rose substantially after 1991 (1991-2009: APC = 9.3, 95% CI: 8.6-10.3, p < 0.001), but has stabilized since 2009. By contrast, the proportion of term-LBW neonates declined markedly between 2002 and 2015 (APC = -4.3, 95% CI: -6.8 to -3.5, p = 0.006) and then remained essentially stable during 2015-2023. The overall increase in SVN was accompanied by a large relative rise in preterm LBW, from 23.7% of all SVN in 1991 to 46.7% in 2023.

Conclusions: The prevalence of preterm and/or LBW newborns in Greece has risen markedly since 1990, reaching a historic peak in 2023. Of particular concern, neonates born both preterm and LBW, the most vulnerable subgroup, were the main drivers of this increase and now constitute the largest share of SVN in the country. These findings underscore the urgent need to elevate the prevention of preterm and LBW births to a national public health priority.

## Introduction

Gestational age and birthweight are the two central determinants of perinatal outcomes. For nearly a century, live births with low birthweight (LBW), defined as a birthweight of < 2,500 g, have been recognized as the classic and most important risk factor for neonatal and infant mortality. Advances in antenatal care led to recognizing preterm birth, defined as delivery before 37 completed weeks, as a key determinant of infant morbidity and mortality [1-5].

Preterm and LBW births are high-priority global health challenges. Despite advances in perinatal medicine, essentially no meaningful progress has been achieved in reducing the prevalence of preterm births worldwide in recent years. In 2020, preterm births still accounted for approximately 10% of all live births, corresponding to an estimated 13.4 million globally [2]. Similarly, the global incidence of LBW births was estimated at 14.7% in 2020 (a total of 19.8 million), showing only modest improvement over the past two decades and still falling far short of internationally established targets [3]. Substantial disparities persist across regions, with the heaviest burden borne by less developed countries.

Infants born preterm or with LBW face a substantially increased risk of mortality and prolonged hospitalization due to severe morbidities affecting multiple organs, including the lungs, central nervous system, and liver. These newborns are also more likely to experience complications in both physical growth and cognitive development. Moreover, prematurity and LBW are now increasingly regarded as chronic conditions, predisposing individuals to a heightened risk of cardiovascular and metabolic diseases later in adulthood [4-10].

The severe and overlapping complications, together with the shared biological, pathological, and socioeconomic determinants, have recently led to the development of a conceptual framework that considers neonates born preterm, with LBW, or small for gestational age (SGA) under the unified term small vulnerable newborns (SVN) [1,11,12]. In Greece, some of the highest rates of preterm and LBW births among developed countries have been reported, following more than three decades of rising trends [13,14]. Accordingly, the objective of this study was to analyze national time trends in the combined burden of preterm and LBW births in Greece since 1980, using this measure as a proxy for the SVN population in the country.

## Materials and methods

Study population

This study utilized publicly available official national data from the Hellenic Statistical Authority [15], derived from birth certificate records. The dataset included all live births in Greece between 1980 and 2023, categorized by gestational age and birthweight.

Inclusion and exclusion criteria

During the study period, a total of 4,585,090 live births with complete information on gestational age and birthweight were analyzed, after excluding 20,679 births (0.45% of the total) with insufficient data.

Study parameters

For each of the 44 years in the period 1980-2023, three categories of SVN were calculated per 100 live births. Specifically, the proportions of neonates born preterm with LBW (preterm-LBW), preterm with birthweight ≥ 2,500 g (preterm-non-LBW), and term (≥ 37 weeks of gestation) with LBW (term-LBW) were estimated. The sum of these three groups represents the annual proportion of SVN in the Greek population, which constituted the primary outcome measure of this study. For the purposes of this analysis, SVN was defined a priori as the combined burden of these three categories.

Statistical analysis

Statistical analyses were performed using Microsoft Excel 2010 (Microsoft Corporation, Redmond, WA, USA). Temporal trends were examined with the Joinpoint Regression Program, version 5.2.0 (National Cancer Institute, Bethesda, MD, USA), which detects points of statistically significant changes in trend. For each segment defined between two joinpoints, the annual percent change (APC) was estimated, allowing a maximum of seven segments. All results are reported with 95% confidence intervals (CI), and statistical significance was defined as p < 0.05.

## Results

**Figure 1:**
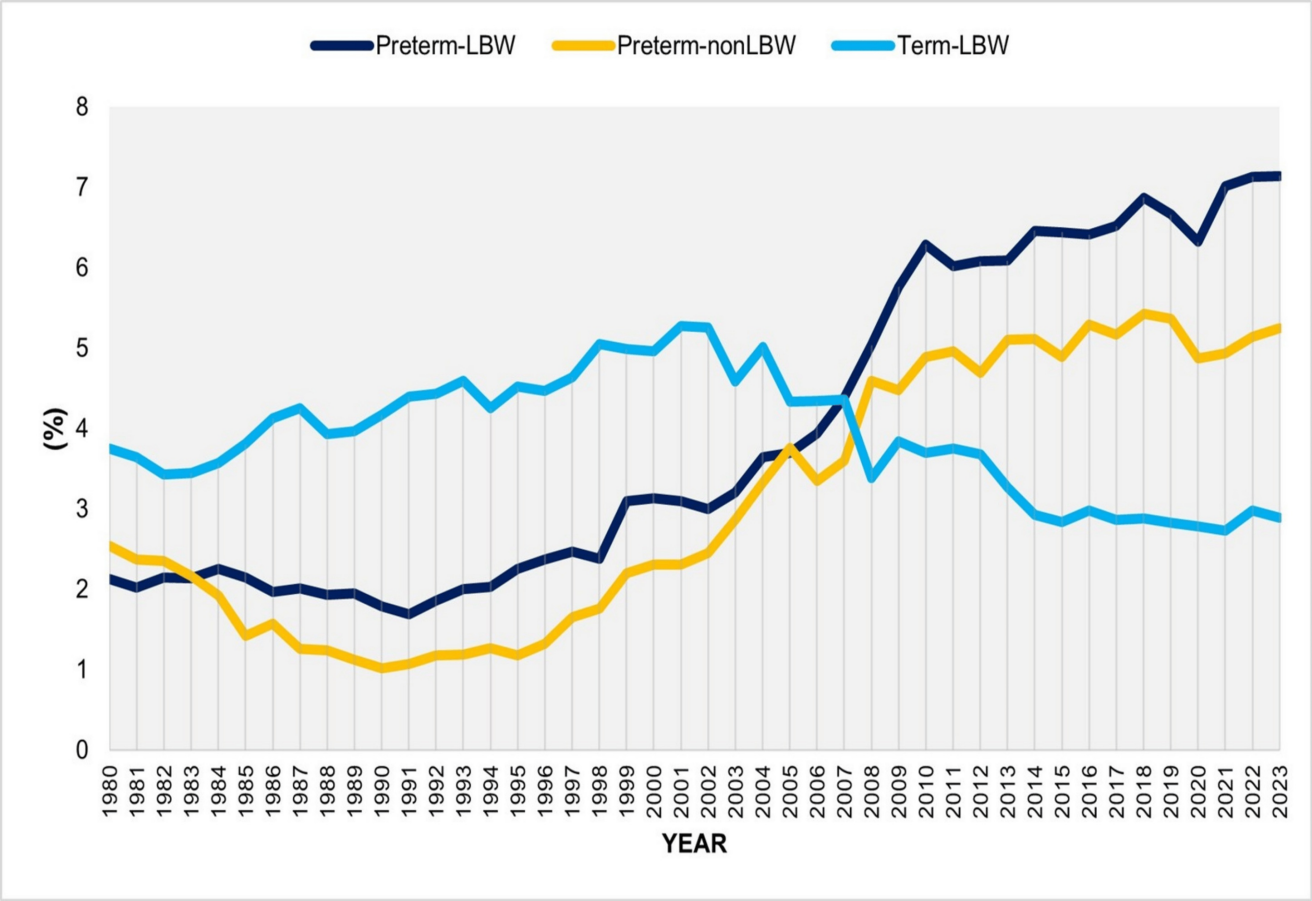
Proportions (%) of preterm-LBW, preterm-non-LBW, and term-LBW births in Greece between 1980 and 2023. LBW: low birthweight

**Figure 2:**
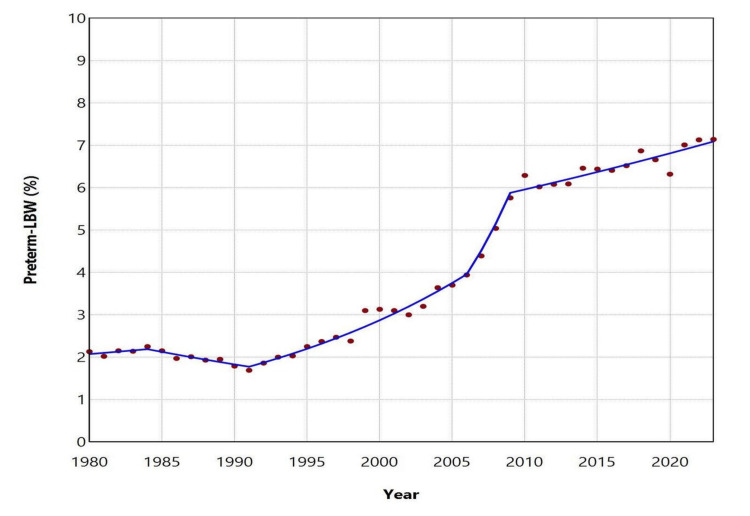
Trends in the proportion (%) of preterm-LBW births in Greece between 1980 and 2023. LBW: low birthweight

The proportion (%) of preterm-LBW births showed no statistically significant changes between 1980 and 1991. From 1991 to 2023, however, a statistically significant upward trend was observed, which was steeper during 1991-2006 (APC = 5.5, 95% CI: 3.8-13.5, p = 0.004) and 2006-2009 (APC = 14.1, 95% CI: 0.4-16.1, p = 0.038), and continued at a slower pace from 2009 to 2023 (APC = 1.3, 95% CI: 0.6-2.2, p = 0.026). Overall, the proportion of preterm-LBW births more than quadrupled, rising from a historic low of 1.69% in 1991 to a historic high of 7.14% in 2023 (Figures 1, 2; Table 1).

**Table 1:** Trends in the proportion (%) of preterm-LBW births between 1980 and 2023. LBW: low birthweight. Trends were calculated using Joinpoint regression analysis.

Segment	Annual percent change	95% confidence interval	P-value
1980-1984	1.4	-1.9 to 7.2	0.394
1984-1991	-3.0	-6.5 to 6.6	0.218
1991-2006	5.5	3.8 to 13.5	0.004
2006-2009	14.1	0.4 to 16.1	0.038
2009-2023	1.3	0.6 to 2.2	0.026

**Figure 3:**
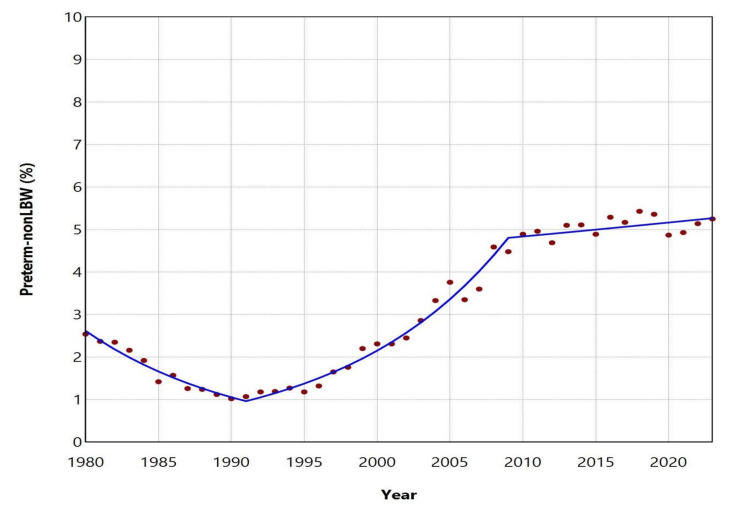
Trends in the proportion (%) of preterm-non-LBW births in Greece between 1980 and 2023. LBW: low birthweight

The proportion (%) of preterm-non-LBW neonates exhibited a clear downward trend between 1980 and 1991 (APC = -8.7, 95% CI: -10.2 to -7.3, p < 0.001). This was followed by a marked upward trend from 1991 to 2009 (APC = 9.3, 95% CI: 8.6-10.3, p < 0.001), after which the proportion stabilized through 2023. The lowest value was recorded in 1990 (1.02%), while the highest was observed in 2018 (5.43%); in 2023, the proportion was 5.25% (Figures 1, 3; Table 2).

**Table 2:** Trends in the proportion (%) of preterm-non-LBW births in Greece between 1980 and 2023. LBW: low birthweight. Trends were calculated using Joinpoint regression analysis.

Segment	Annual percent change	95% confidence interval	P-value
1980-1991	-8.7	-10.2 to -7.3	< 0.001
1991-2009	9.3	8.6 to 10.3	< 0.001
2009-2023	0.7	-0.4 to 1.6	0.207

**Figure 4:**
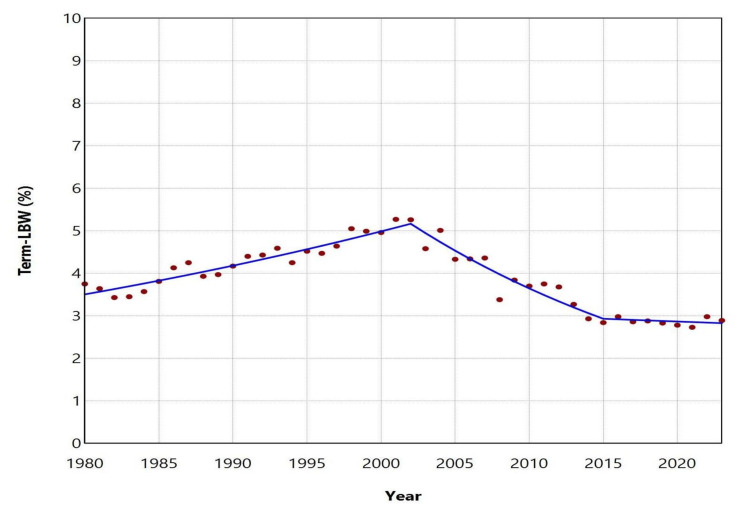
Trends in the proportion (%) of term-LBW births in Greece between 1980 and 2023. LBW: low birthweight

In contrast, the proportion (%) of term-LBW neonates showed an initial upward trend between 1980 and 2002 (APC = 1.8, 95% CI: 1.4-2.2, p = 0.002), followed by a pronounced decline from 2002 to 2015 (APC = -4.3, 95% CI: -6.8 to -3.5, p = 0.006). Thereafter, the trend remained essentially stable during 2015-2023. The proportion ranged from a peak of 5.27% in 2001 to a low of 2.73% in 2021, reaching 2.89% in 2023 (Figures 1, 4; Table 3).

**Table 3:** Trends in the proportion (%) of term-LBW births in Greece between 1980 and 2023. LBW: low birthweight. Trends were calculated using Joinpoint regression analysis.

Segment	Annual percent change	95% confidence interval	P-value
1980-2002	1.8	1.4 to 2.2	0.002
2002-2015	-4.3	-6.8 to -3.5	0.006
2015-2023	-0.4	-2.1 to 5.0	0.718

**Figure 5:**
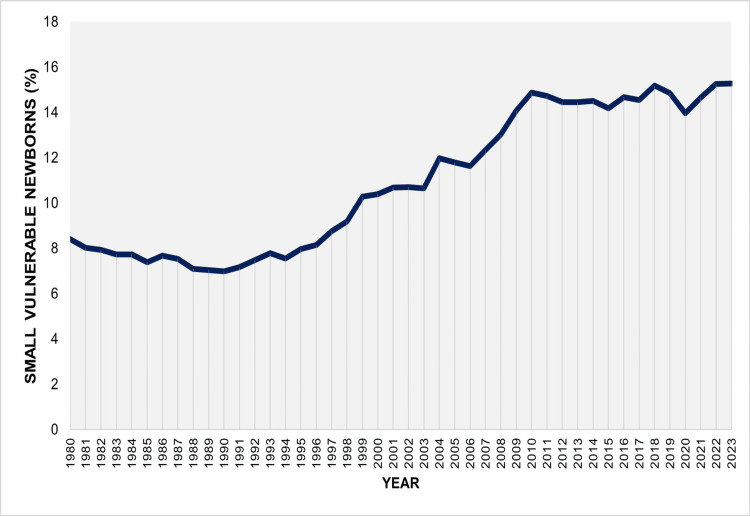
Proportion (%) of small vulnerable newborns in Greece between 1980 and 2023.

**Figure 6:**
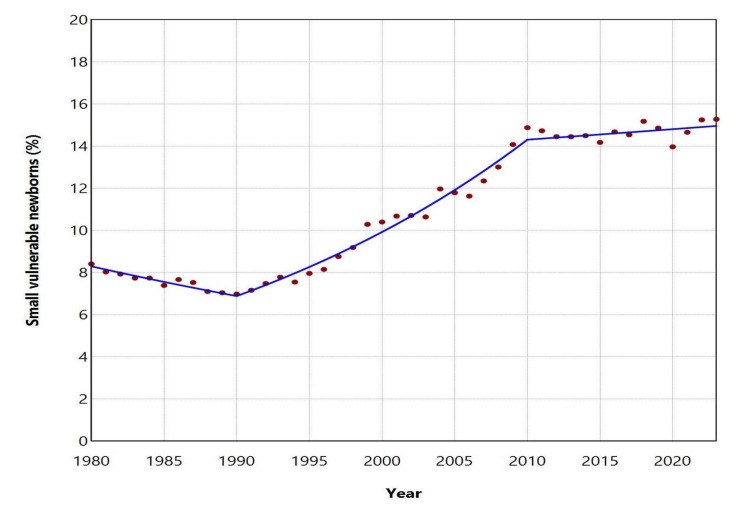
Trends in the proportion (%) of small vulnerable newborns in Greece between 1980 and 2023.

Over the entire period 1980-2023, a total of 488,832 neonates were recorded as being preterm, LBW, or both, corresponding to 10.66% of all live births. This percentage declined significantly during the first decade, from 8.41% in 1980 to an all-time low of 6.98% in 1990 (APC = -1.8, 95% CI: -2.7 to -1.1, p < 0.001). However, a marked deterioration followed over the subsequent two decades (1990-2010: APC = 3.7, 95% CI: 3.5 to 4.0, p < 0.001), during which this percentage more than doubled, reaching 14.88% in 2010. In the most recent period (2010-2023), the proportion of affected neonates among all live births showed a non-significant upward trend (APC = 0.3, 95% CI: -0.2 to 0.8, p = 0.178) (Figures 5, 6; Table 4).

**Table 4:** Trends in the proportion (%) of small vulnerable newborns in Greece between 1980 and 2023. Trends were calculated using Joinpoint regression analysis.

Segment	Annual percent change	95% confidence interval	P-value
1980-1990	-1.8	-2.7 to -1.1	< 0.001
1990-2010	3.7	3.5 to 4.0	< 0.001
2010-2023	0.3	-0.2 to 0.8	0.178

**Figure 7:**
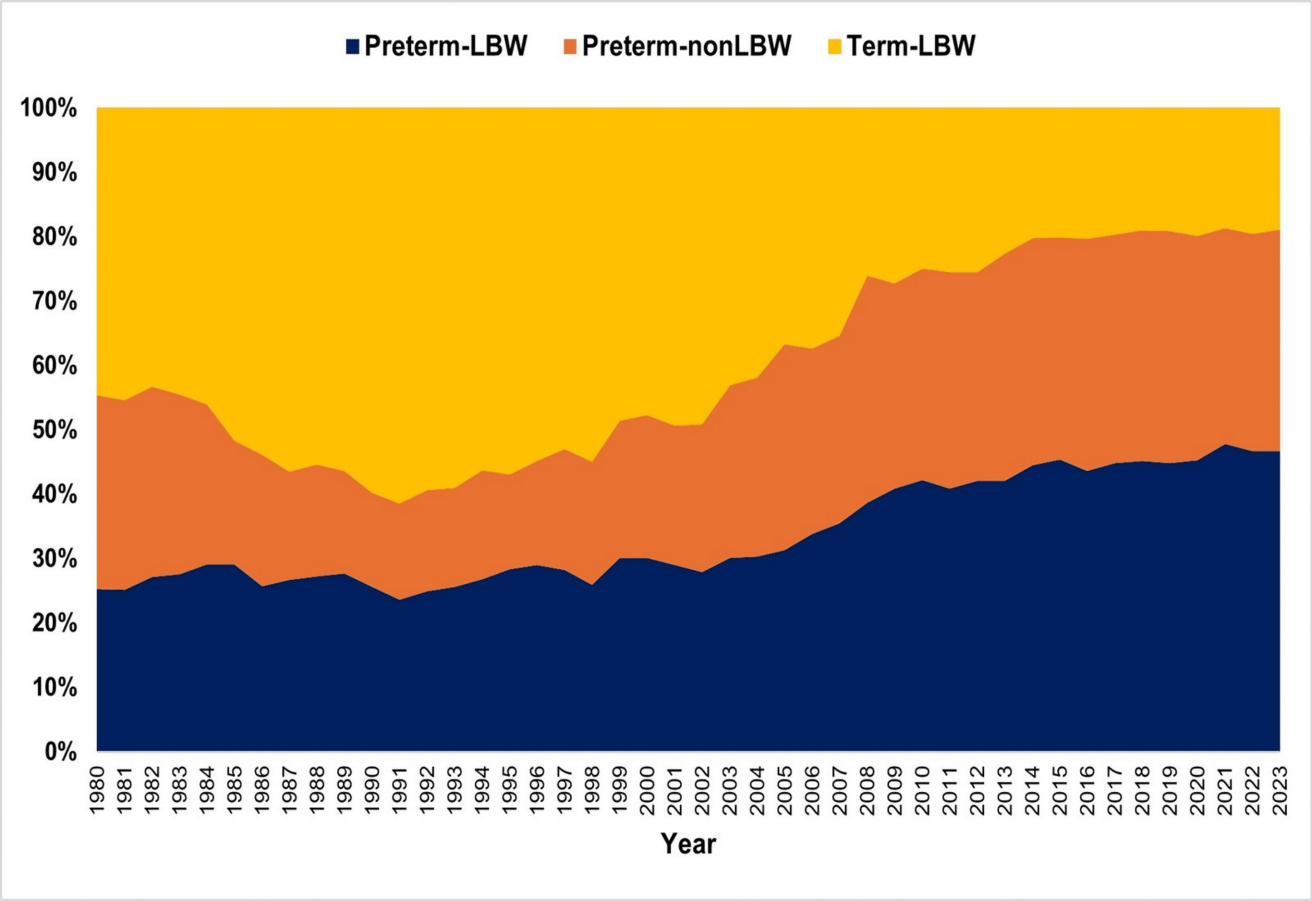
Distribution (%) of preterm-LBW, preterm-non-LBW, and term-LBW among small vulnerable newborns in Greece between 1980 and 2023. LBW: low birthweight

In 2023, Greece recorded its highest-ever proportion of SVNs at 15.28%. The distribution across the three subcategories was as follows: preterm-LBW, 46.7%; preterm-non-LBW, 34.4%; and term-LBW, 18.9%. Overall, prematurity accounted for 81.1% of the total SVN burden. By comparison, in 1991, only 38.6% of SVNs were preterm, with the distribution being preterm-LBW, 23.7%; preterm-non-LBW, 15.0%; and term-LBW, 61.4%. While term-LBW represented the most common SVN subgroup from 1980 to 2006, since 2007, the preterm-LBW subgroup has become the most frequent (Figures 1, 7).

## Discussion

This comprehensive analysis of official national data highlights the substantial burden of SVN in the Greek population. The prevalence of SVN in Greece rose sharply during the 1990s and 2000s. Since 2010, the upward trend has leveled off to borderline non-significant levels; however, in 2023, the proportion reached a historic peak, with preterm and/or LBW neonates accounting for 15.28% of all live births in the country.

A subgroup analysis was conducted separately for the three categories derived from the combined assessment of preterm and LBW births. Both the preterm-LBW and preterm-non-LBW proportions increased significantly between 1991 and 2009. During 2009-2023, the preterm-non-LBW proportion stabilized, whereas the preterm-LBW proportion continued to rise significantly through 2023. In contrast, the term-LBW proportion showed a steady increase from 1980 to 2002, followed by a declining trend and stabilization after 2015. Overall, the post-1990 upward trajectory of total SVN percentage was accompanied by major shifts in subgroup composition, with an increasing contribution from preterm categories and a marked reduction in term-LBW neonates.

From a more concise perspective, in the present analysis, SVN arises from the combination of preterm neonates (with or without LBW) and those who, despite reaching 37 weeks of gestation, were born with a birthweight < 2,500 g. This latter category of term-LBW neonates is regarded as a proxy for SGA infants born at term. In Greece, the proportion of SVNs that were preterm soared from 38.6% in 1991 to 81.1% in 2023. The precise reasons for the decline in the proportion of term-LBW neonates remain unclear and warrant further investigation. Internationally, an upward trend in the birthweight of term neonates has been reported [16], which may be linked to improvements in antenatal care and overall living standards. It is well established that the relative contributions of preterm and term SVN vary considerably across countries, largely reflecting differences in economic development and socioeconomic conditions. In low- and middle-income settings, growth-restricted term infants account for a greater share of SVN, as fetal growth is more strongly constrained by factors such as maternal nutritional status and pregnancy-related morbidity [17]. In Greece, however, close antenatal surveillance may contribute to a higher frequency of clinical interventions, such as early induction of labor or elective cesarean delivery in cases of SGA, which in turn could explain the relative increase in preterm SVN cases.

In its original conception, SVN referred to preterm neonates plus term-SGA infants, thereby avoiding the dichotomous consideration of LBW [1]. However, defining SVN based on SGA status also carries limitations, since SGA infants do not coincide with those experiencing fetal growth restriction (FGR), the group at truly elevated risk of immediate and long-term sequelae [18]. In the present analysis, the SVN population was defined as the sum of preterm neonates and those born at term with LBW, representing a slightly modified operational definition adopted due to the lack of available data on SGA status. Term-LBW is generally considered a reasonably accurate surrogate for term-SGA at the population level [3]. Nevertheless, the prevalence of SVN reported here is likely an underestimation of the true burden, as the term-SGA group extends beyond term-LBW, with a proportion of term-SGA infants not classified as LBW [1].

A particularly noteworthy finding of this study is the relative increase in preterm-LBW neonates. This subgroup is the only category among SVN that has continued to show an upward trend since 2009 and now represents the largest proportion of SVN in the country. Preterm-LBW infants face the highest risks of mortality and morbidity. Conceptually, this group includes both preterm-SGA neonates, recognized as the most vulnerable of all, as well as the smaller preterm-appropriate for gestational age (AGA) infants, who also remain at elevated risk compared with their term non-SGA counterparts.

Global estimates suggest that term-SGA newborns account for approximately 62% of all SVN, while about 38% are preterm, with substantial variability across regions. Around 3% of newborns are classified as term-non-LBW-SGA. Across European countries, the total prevalence of SVN (preterm plus term-SGA) ranges from 7.7% in Estonia to 12.1% in Scotland, considerably lower than the levels observed in Greece. Preterm-SGA infants, who represented less than 1% of total live births, carried the highest risk of neonatal mortality, followed by preterm-AGA and preterm-LGA neonates, which showed comparable risks; in comparison, term-SGA infants had a lower risk [17,19]. In an international study of six developing countries, the distribution of SVN was 26.7% preterm-LBW, 34.2% preterm-non-LBW, and 39.1% term-LBW, remarkably higher proportions of term-LBW than those observed in the Greek population [20].

In this study, a comprehensive analysis of more than four decades of data in Greece revealed a dramatic increase in the combined prevalence of preterm and LBW neonates since 1990, reaching a historic peak in 2023. Assessing the aggregate proportion of infants born preterm, LBW, or both underscores the substantial burden of SVN in Greece, with the exception of the term-non-LBW-SGA group, for which detailed national birthweight distribution data were unavailable. This study has certain limitations. First, they relate to the challenges of accurately determining gestational age for the classification of prematurity, whether based on the last menstrual period or ultrasound assessment [13]. Second, the study is descriptive in nature, designed to document trends in the occurrence of preterm and/or LBW newborns in Greece, without addressing the underlying causes of these trends. Further investigations linking these proportions to factors such as maternal age, multiple gestations, infant sex, and various socioeconomic and biological determinants could provide valuable insights into the parameters shaping the country’s perinatal landscape. Finally, although the present analysis estimated the prevalence of SVN using available national data on gestational age and birthweight, future research incorporating distinctions between SGA and non-SGA infants may provide more informative insights.

This analysis carries important implications for public health and perinatal medicine. The rising prevalence of preterm and/or LBW births continues to impede progress in neonatal and infant mortality rates in Greece [21]. These infants also face an increased risk of developmental and cardiometabolic disorders later in life, often manifesting at younger ages [6,9,10]. The exceptionally high levels of SVN highlight the urgent need for targeted strategies, with the prevention of preterm and LBW births elevated to a national health priority. Recognition of the magnitude of the problem is essential, given its considerable health and economic costs, both for immediate neonatal care and for long-term outcomes. Strengthened systems for recording and monitoring pregnancy and birth outcomes, together with effective preventive measures and comprehensive antenatal care, particularly for high-risk pregnancies, are imperative [22,23].

## Conclusions

This nationwide analysis demonstrates a sustained upward trend in the combined prevalence of preterm and/or LBW births in Greece since 1990. Of particular concern, infants who were both preterm and LBW, the most vulnerable subgroup, emerged as the main drivers of the recent increase and now constitute the largest share of SVN in the country. These findings underscore the urgent need to prioritize the prevention of preterm and LBW births as a national health priority. Strengthened surveillance systems, enhanced social support, improved access to obstetric care, and the implementation of targeted, evidence-based interventions are essential to reverse adverse perinatal trends in Greece.
